# Factor structure of medical students’ attitudes towards psychiatry: findings from a nationally representative sample from Sri Lanka

**DOI:** 10.1192/bjo.2021.360

**Published:** 2021-06-18

**Authors:** Anuradha Baminiwatta, Miyuru Chandradasa, Dileepa Ediriweera, Shavindra Dias

**Affiliations:** 1 Department of Psychiatry, Faculty of Medicine, University of Kelaniya; 2 Department of Psychiatry, Faculty of Medicine, University of Kelaniya, Ragama, Sri Lanka; 3 Computer Centre, Faculty of Medicine, University of Kelaniya; 4 Department of Psychiatry, Faculty of Medicine, University of Peradeniya

## Abstract

**Aims:**

The aim of this study was to examine the factor structure of attitudes towards psychiatry among medical students by using the ‘Attitude towards psychiatry-30’ (ATP-30) scale, which is one of the most widely used psychometric tools in assessing medical students’ attitudes regarding psychiatry. We also aimed to explore the possible existence of meaningful subscales in the ATP-30 scale.

**Method:**

Secondary data from a survey of 743 final-year medical students from nine medical schools in Sri Lanka were subjected to factor analysis. Models based on empirical evidence were tested with Confirmatory Factor Analysis (CFA) for model fit using Comparative Fit Index (CFI), Tucker Lewis Index (TLI), root mean square error of approximation (RMSEA) and Chi square. To explore the underlying latent structure of the scale, Exploratory Factor Analysis (EFA) with oblique (i.e. Promax) rotation was employed. Horn's parallel analysis and goodness-of-fit statistics for a series of EFA models tested with different numbers of factors were used in determining the number of factors to retain. Items conceptually external to the emerging factors or with factor loadings less than 0.4 were discarded. Gender invariance of the final model was tested by configural, metric and scalar invariance. Internal consistency of subscales was assessed using McDonald's omega (ω).

**Result:**

Three models based on literature (one-, five-, and eight- factor) were disproved by CFA. EFA revealed a six-factor solution, encompassing 18 out of the 30 items, to be the most theoretically meaningful factor structure. This six-factor model was affirmed by a CFA (CFI = 0.94, TLI = 0.92, RMSEA = 0.036). These factors were, namely, ‘the image of psychiatrists’, ‘psychiatric patients and mental illness’, ‘efficacy of treatment’, ‘psychiatric teaching’, ‘career choice’, and ‘psychiatry as an evidence-based discipline’. This six-factor solution was invariant across gender. ‘The image of psychiatrists’ appeared to be the most salient factor, and formed the most consistent subscale (ω = 0.71). The internal consistencies of the other subscales were modest (ω = 0.55–0.67). The overall 18-item scale showed good internal consistency (ω = 0.78).

**Conclusion:**

Our findings provide evidence of a multi-dimensional structure in medical students’ attitudes towards psychiatry, endorsing six meaningful subscales of the ATP-30. Future researchers and educators can utilize these subscales in identifying specific attitudinal domains which are more closely associated with students’ future choice of a career in psychiatry, and also in identifying specific areas where attitudes are more stigmatized, so that appropriate interventions can be incorporated into the undergraduate psychiatric curriculum.

